# Exploration of plasma adiponectin, leptin, and *COMT* genotype on blood pressure among women who are post-menopause

**DOI:** 10.1017/jns.2023.75

**Published:** 2023-09-19

**Authors:** Lauren Green, Laura Byham-Gray, Mindy Kurzer, Hamed Samavat

**Affiliations:** 1 School of Health Professions, Department of Clinical and Preventive Nutrition Sciences, Rutgers University, Newark, NJ, USA; 2 Department of Food Science and Nutrition, University of Minnesota, St. Paul, MN, USA

**Keywords:** Adiponectin, Blood pressure, *COMT* genotype, Diastolic blood pressure, Leptin, Systolic blood pressure

## Abstract

Research suggests that adiponectin, leptin, and genetic polymorphisms such as catechol-*O*-methyltransferase (*COMT*) genotype may play an integral role in blood pressure status and thereby cardiovascular health. This is an area especially important for women who are post-menopause; however, the current literature investigating these associations is limited. This study was a cross-sectional secondary analysis of baseline data (*N* 237) from the Minnesota Green Tea Trial (MGTT). The current study explored the relationships between plasma adiponectin, leptin, and *COMT* genotype on blood pressure measures. Plasma adiponectin and leptin were obtained after an overnight fast of at least 10 h and were measured by the radioimmunoassay method. The relationships were analysed using multiple linear regression after adjusting for potential confounders. Effect modifications by age, body mass index (BMI) category, blood pressure category, antihypertensive medication use, and *COMT* genotype were also investigated. The majority of participants were non-Hispanic (97⋅9 %) and Caucasian (94⋅9 %). Mean (sd) age and BMI were 60⋅7 (5⋅0) years and 28⋅2 (2⋅9) kg/m^2^, respectively. After adjustment for confounding variables, neither plasma adiponectin, plasma leptin nor *COMT* genotype was associated with systolic or diastolic blood pressure measures. The results of stratified analyses also did not reveal any significant interactions or associations. Based on the findings of this study, which utilised more rigorous statistical methods than previous research, neither adiponectin, leptin nor *COMT* genotype play a role in blood pressure measures in women who are post-menopause.

## Introduction

Changes that occur as a result of menopause such as withdrawal of oestrogen, increased waist circumference, and other biochemical changes may put women at increased risk of cardiovascular disease (CVD).^(1,2)^ CVD is the leading cause of death of women in the United States; linked to one in every five deaths.^(3)^ The aetiology of CVD is multifactorial and has long been associated with comorbid conditions such as diabetes, obesity, and hypertension.^(4–6)^ Novel research, however, suggests that hormones, such as adiponectin and leptin, and genetic polymorphisms such as catechol-*O*-methyltransferase (*COMT*) genotype may also play an integral role in the development of the disease as well.^(7–14)^

Adiponectin is an adipokine or fat-derived hormone that contributes to many important actions in the body and has also been found to play a role in blood pressure through its role in endothelial function, nitric oxide production, and its support for the action of anti-inflammatory macrophages.^(15,16)^ An inverse relationship between hypoadiponectinemia and hypertension has been previously reported in both men and women who were hypertensive.^(16)^ This relationship may be attributed to adiponectin's effects on the vascular system or implications related to fat or glucose metabolism.^(16)^ While these findings are interesting, gender differences in plasma adiponectin levels also exist that should also be accounted for.^(13,16–18)^ For example, the majority of research that has specifically explored the relationship between adiponectin and blood pressure in women who are post-menopause does not support the reported findings of an inverse relationship.^(16,19–23)^

Similar to adiponectin, leptin is an adipokine that may also impact blood pressure status due to its effect on sympathetic nervous system activation at the renal level.^(14,24–28)^ Many studies have demonstrated a positive correlation between leptin and blood pressure.^(24,29–32)^ Also, similar to adiponectin, gender differences exist with women's leptin levels generally being higher than that of men's leptin levels.^(33)^ Available research exploring the association between leptin and blood pressure in women who are post-menopause suggests that there is a relationship in those ranging from 50 to 68 years old.^(20,22,34)^ Interestingly, however, leptin has been primarily associated with systolic blood pressure (SBP) in this population.^(10,14,20,22,34)^

Catechol-*O*-methyltransferase or *COMT* is an enzyme that is involved in the metabolism of catecholamines, oestrogens, and catechins.^(7,8,35)^ *COMT* genotype is polymorphic; an individual's *COMT* genotype may impact COMT enzymatic activity.^(8,9,35,36)^ Evidence exists to support a potential relationship between *COMTA/A* (low activity) genotype and higher SBP, however, to date, no study has explored this association specifically in women who are post-menopause.^(9)^

Current evidence exploring the associations between adiponectin, leptin, and *COMT* genotype on blood pressure in women who are post-menopause is limited. Understanding more about these novel factors can perhaps help to identify those potentially at risk at an earlier time which could also help with earlier monitoring and potentially contribute to the prevention of CVD as well. This cross-sectional study, therefore, contributes to and expands upon the existing literature with a primary aim to determine the associations between plasma adiponectin and leptin levels and blood pressure among women who were post-menopause. We hypothesised that there would be an inverse relationship between adiponectin and blood pressure while there would be a positive association between leptin and blood pressure in women who were post-menopause. In addition, a secondary and exploratory aim of this study was to determine the associations between *COMT* genotype and blood pressure among the same sample. We hypothesised that there would be a positive association between *COMTA/A* genotype and blood pressure while there would be no association between *G/G* and *G/ACOMT* genotypes and blood pressure in women who are post-menopause.

## Materials and methods

### Study design

This study was a cross-sectional study, which was completed as a secondary analysis of existing data from the parent study, the Minnesota Green Tea Trial (MGTT).^(37)^ The MGTT was a randomised double-blind placebo-controlled clinical trial in which the participants were randomly assigned to a green tea extract supplement or placebo group.^(37)^ The primary aim of the parent study was to investigate the effect of the consumption of green tea extract on biomarkers of breast cancer risk in a group of women who were post-menopause, between 50 and 70 years of age, and who were at high risk for breast cancer due to having heterogeneously (51–75 % glandular) or extremely (>75 % glandular) dense breasts.^(37)^

### Study procedures

The dataset for this study was a subgroup of the population recruited for the MGTT. Only the women who had plasma adiponectin and leptin values available for the baseline visit were included (*N* 237) in the sample.^(38)^ The MGTT was an interventional study where a green tea extract supplement was consumed by the participants; therefore, baseline data was used to exclude any potential effect that the intervention may have had on the variables of interest for this study.^(37,38)^ Potential participants in the MGTT were identified by study staff reviewing routine screening mammogram reports.^(37)^ Participants were then contacted to identify interest and further included in the MGTT based on the inclusion and exclusion criteria detailed in the following.^(37)^ Participants were included in the MGTT if they were generally healthy women, were post-menopause between 50 and 70 years old with heterogeneously (51–75 % glandular) or extremely (>75 % glandular) dense breasts and required to reside in or near Minnesota during the study.^(37)^ Participants also gave informed consent as part of the inclusion criteria of the MGTT.^(37)^ Participants were excluded if they consumed more than one cup of green tea per week, had Hepatitis B or C, elevated liver enzymes (>1⋅5 times the upper limit of normal), a history (within 6 months from the start of the trial) or use of menopausal hormone therapy during screening, a previous diagnosis of breast or ovarian cancers, a history of any other cancers within past 5 years, proliferative breast disease, breast implants, BMI <18⋅5 or >40 kg/m^2^, were taking methotrexate or etanercept, were involved in a weight gain or loss programme, had a weight change of greater than 10 pounds during the year prior to recruitment, had an intake of more than seven alcoholic drinks per week, or smoked cigarettes or consumed other tobacco products.^(37)^ The present study utilised the same inclusion and exclusion criteria as the MGTT with two small additions based on the specific outcomes of interest for this study.^(37)^ While there were no additional exclusions added for the execution of this study, participants with available plasma adiponectin and leptin data at baseline of the MGTT was an additional inclusion criterion for this study.

### Blood pressure

The outcome of interest for this study was blood pressure measures including both SBP and diastolic blood pressure (DBP). As part of the MGTT, blood pressure was measured using an automated digital vital sign monitor (Philips Healthcare, Eindhoven, The Netherlands).^(37)^ Participants were asked to sit with legs and ankles uncrossed following resting and relaxing for 5 min. If the blood pressure value was above 120 mmHg for SBP or 80 mmHg for DBP, it was measured again to confirm the accuracy. Additionally, blood pressure categories (normotensive as SBP < 120 mmHg and DBP < 80 mmHg; elevated blood pressure as SBP = 120–129 mmHg and DBP < 80 mmHg; and hypertensive as SBP ≥ 130 mmHg or DBP ≥ 80 mmHg) as defined by the American Heart Association (AHA) and the American College of Cardiology's (ACC) 2017 guidelines were used to assess the data.^(39)^ As the parent study was completed prior to 2017 classifications of the AHA and ACC (normotensive: <140/90 *v.* hypertensive: >140/90), further stratified analysis was conducted to explore the differences that may exist in the findings based on the different blood pressure guidelines.^(39)^

### Plasma adiponectin and plasma leptin

Plasma adiponectin and leptin levels were measured in blood samples from the MGTT that were obtained after an overnight fast of at least 10 h.^(38)^ Whole blood samples were separated into plasma and serum and stored at –80°C until the time of analysis. Adiponectin and leptin concentrations were quantified in plasma utilising the radioimmunoassay method using EMD Millipore kits (inter-assay coefficient of variation (CV): leptin = 7⋅1 %, adiponectin = 8⋅9 %; intra-assay CV: leptin = 6⋅6 %, adiponectin = 7⋅4 %).^(38)^

### COMT genotype

*COMT* genotype was determined from the MGTT using whole blood samples obtained from non-fasting blood draws and analysed by the University of Minnesota Genomics Center.^(37,38)^ DNA was extracted using the Qiagen DNAeasy Blood and Tissue Kit method (Qiagen Inc., Gaithersburg, MD).^(37,38)^ To determine the *COMT* polymorphism, a TaqMan assay was developed using a TaqMan PCR Core Reagents Kit (Applied Biosystems).^(37,38)^ Additionally, as a quality control for each PCR run, Coriell cells with a known *COMT* genotype were used.^(38)^ The data are presented and analysed by the activity of the relevant *COMT* genotypes: low (*A/A*), intermediate (*G/A*), and high (*G/G*) activity *COMT* genotypes.

Other variables used in this study were body mass index (BMI), waist-to-hip ratio (WHR), sodium, caffeine and alcohol consumption, physical activity, age, antihypertensive medication use, and vitamin and mineral supplement use. Values needed to calculate BMI and WHR were obtained by trained clinic staff in the MGTT. BMI numerical values and the corresponding CDC categories (i.e. normal: 18⋅5–24⋅9 kg/m^2^, overweight: 25–29⋅9 kg/m^2^, and obese: ≥30 kg/m^2^) were used to assess the BMI data.^(40)^ Diet component and energy intake data were obtained using the Diet History Questionnaire 1 (DHQ1) in the MGTT.^(37,38)^ The DHQ1 was developed and validated by the National Institutes of Health (NIH) and completed by the participants of the MGTT at the baseline.^(41)^ Using data from the DHQ1, average food and nutrient intake was estimated using DietCalc software created by the National Cancer Institute.^(38)^ Physical activity was assessed by a health history questionnaire (HHQ) in the MGTT.^(37,38)^ The HHQ asked participants questions about physical activity including the type, frequency, and duration of activity.^(37,38)^ The average hours of each activity per week were multiplied by its metabolic equivalent (MET) hour equivalent to produce MET-hours for each activity for each participant weekly.^(37,38)^ Age and antihypertensive medication use as well as vitamin and mineral supplement use were also obtained from the HHQ.^(37,38)^

### Statistical methods

Descriptive statistics were reported for all variables. Histograms and boxplots were used to assess the normality of the data to identify any extreme outliers. Scatterplots were also used to visually assess the linearity of the relationships between the variables. Bivariate analyses were performed with ANOVA and Pearson's correlation to test for potential unadjusted relationships between predictor variables (plasma adiponectin, plasma leptin, and *COMT* genotype) and outcome variables (SBP and DBP). These analyses and clinical relevance were considered to identify potential confounding variables that would be covariates used in the adjusted models and included age, BMI, WHR, energy intake, energy, sodium, caffeine and alcohol consumption, physical activity, and antihypertensive medication use. Different linear regression models were constructed to explore associations between adiponectin, leptin, blood pressure measures, and *COMT* genotype. The models included the crude analyses with no adjustment, and models adjusted for age, BMI and physical activity as model 1; and model 2 with adjustment for age, BMI, WHR, physical activity, energy intake, sodium intake, caffeine intake, total drinks of alcohol per week, and adiponectin or leptin where appropriate. To explore the association between *COMT* genotype and blood pressure measures, a model was built and adjusted for age. In addition, we conducted subgroup analyses based on age, BMI category and blood pressure category, antihypertensive medication use and *COMT* genotype, adiponectin and leptin where appropriate. *P-*values for the interaction of age, BMI, *COMT* genotype, blood pressure category, current antihypertensive medication use, and adiponectin and leptin, where appropriate, on the blood pressure measures were calculated by a generalised linear model while also adjusting for age, BMI, and physical activity where appropriate.

Since this was a secondary analysis of available data, a *post hoc* power analysis was conducted on the primary hypothesis using linear multiple regression, fixed model function in G*power 3.1.9.7 version.^(42)^ Using an alpha level of 0⋅05, a relatively small effect size of 0⋅05 with eight covariates, and an existing sample size of 237 this study had more than 80 % statistical power. All statistical analyses were performed using SPSS version 27.0 (SPSS Inc, Chicago, IL). *P*-values ≤0⋅05 were considered statistically significant.

## Results

Data were utilised for 237 participants from the MGTT for this study (Table 1). The mean (sd) age of the sample was 60⋅7 (5⋅0) years and the mean (sd) of BMI was 28⋅1 (2⋅9) kg/m^2^. The majority of participants were non-Hispanic (97⋅9 %), Caucasian (94⋅9 %), and had never smoked (67⋅9 %). More than 71 % of participants reported not using antihypertensive medications. Blood pressure categorisation revealed that 32⋅9 % of the sample population were categorised as normotensive, 20⋅7 % were categorised as having elevated blood pressure, and 46⋅4 % were categorised as hypertensive.

**Table 1.** Selected demographic and clinical characteristics of the sample (*N* 237)

Characteristic	Mean ± sd or median (IQR)	*n* (%)
Age (years)^a^	60⋅7 ± 5⋅0	
Race^a^		
Caucasian		225 (94⋅9)
Asian		2 (0⋅8)
Black		7 (3⋅0)
Others		2 (0⋅8)
Unknown		1 (0⋅4)
Ethnicity		
Hispanic		1 (0⋅4)
Non-Hispanic		232 (97⋅9)
Unknown		4 (1⋅7)
Education		
High school or less		22 (9⋅3)
College degree or some college		140 (59⋅1)
Masters/PhD/Professional		74 (31⋅2)
Unknown		1 (0⋅4)
Smoking status^b^		
Never		161 (67⋅9)
Former		75 (31⋅6)
Unknown		1 (0⋅4)
Current antihypertensive medication use		
Yes		67 (28⋅3)
No		170 (71⋅7)
Current vitamin and mineral supplement use		
Yes		38 (16⋅0)
No		199 (84⋅0)
Body mass index (kg/m^2^)		
Normal (18⋅5–24⋅9)		26 (11⋅0)
Overweight (25⋅0–29⋅9)		151 (63⋅7)
Obese (≥30)		60 (25⋅3)
Waist-to-hip ratio (WHR)	0⋅86 (0⋅85, 0⋅87)	
Total physical activity (MET-h/week)^c^	25⋅5 (10⋅9, 49⋅4)	
Energy intake (kcal)^d^	1488⋅4 (1412⋅5, 1564⋅4)	
Sodium (mg)	2135⋅4 (1583⋅0, 2634⋅6)	
		
Caffeine (mg)	305⋅8 (78⋅2, 610⋅3)	
Alcoholic drinks (/week)	1⋅3 (0⋅5, 4⋅4)	
*COMT* genotype^a^		
*A/A* (Low activity)		75 (31⋅6)
*G/A* (Intermediate activity)		110 (46⋅4)
*G/G* (High activity)		52 (21⋅9)
Systolic blood pressure (mmHg)^a^	125⋅5 ± 12⋅8	
Diastolic blood pressure (mmHg)^a^	75⋅0 ± 9⋅4	
Blood pressure category^e^		
Normotensive		78 (32⋅9)
Elevated blood pressure		49 (20⋅7)
Hypertensive		110 (46⋅4)
Plasma adiponectin (μg/ml)	6⋅2 (4⋅3, 9⋅7)	
Plasma leptin (μg/l)	31⋅4 (21⋅2, 45⋅8)	

*COMT*, catechol-*O*-methyltransferase; DBP, diastolic blood pressure; IQR, interquartile range; MET, metabolic equivalents; SBP, systolic blood pressure.

a Data reported as mean ± standard deviation.

b Percentages may not add up to 100 % due to rounding.

c Data missing for two participants.

d Data missing for one participant.

e Normotensive is defined as SBP < 120 mmHg and DBP < 80 mmHg; elevated blood pressure as SBP = 120–129 mmHg and DBP < 80 mmHg; and hypertensive as SBP ≥ 130 mmHg or DBP ≥ 80 mmHg.

Associations between adiponectin, leptin, and blood pressure measures are displayed in Table 2. There was a significant positive association between leptin and SBP in the crude analysis (*β* = 0⋅128, *P* = 0⋅049), however, the statistical significance was lost in adjusted analyses. Crude analyses also revealed that there was not a significant association between adiponectin with either SBP or DBP or between leptin and DBP. To further assess the association, two adjusted models were utilised, one in which BMI and physical activity were controlled for and another in which BMI, WHR, physical activity, energy intake, sodium intake, caffeine intake, total drinks of alcohol per week, and adiponectin or leptin where appropriate were controlled for. The results of these analyses remained unchanged from the crude analysis with the exception of leptin and SBP, as discussed.

**Table 2.** Associations between circulating concentrations of adiponectin and leptin and blood pressure measures (*n* 237)^a^

	Crude model^b^			Adjusted model^c^			Fully adjusted model^d^		
	Β	95 % CI	*P-*value	Β	95 % CI	*P-*value	*β*	95 % CI	*P-*value
Systolic blood pressure									
Adiponectin	−0⋅068	−0⋅307, 0⋅171	0⋅577	−0⋅015	−0⋅245, 0⋅214	0⋅896	0⋅001	−0⋅217, 0⋅219	0⋅995
Leptin	0⋅070	0⋅000, 0⋅140	0⋅049	0⋅036	−0⋅430, 0⋅115	0⋅365	0⋅035	−0⋅040, 0⋅111	0⋅358
Diastolic blood pressure									
Adiponectin	0⋅022	−0⋅154, 0⋅198	0⋅806	0⋅053	−0⋅124, 0⋅230	0⋅554	0⋅067	−0⋅103, 0⋅237	0⋅430
Leptin	0⋅026	−0⋅026, 0⋅077	0⋅330	−0⋅006	−0⋅067, 0⋅055	0⋅845	−0⋅005	−0⋅064, 0⋅054	0⋅859

CI, confidence interval.

a Data missing for one participant for waist-to-hip ratio (WHR) and for two participants for physical activity.

b Crude model: no adjustments were made.

c Adjusted model 1: adjusted for age, body mass index, and physical activity.

d Adjusted model 2: adjusted for covariates in model 1 as well as sodium, caffeine, total drinks of alcohol per week, energy intake, WHR, antihypertensive medication use, and adiponectin or leptin where appropriate.

Associations between adiponectin and leptin and blood pressure measures were explored further through stratified analyses exploring potential interactions of age, BMI, *COMT* genotype, blood pressure category, and current blood pressure use with adiponectin and leptin and DBP. Tests of interaction revealed no significant interaction between adiponectin and leptin and SBP or DBP measures with age, BMI, *COMT* genotype, blood pressure category, and current antihypertensive medication use (see Supplementary Tables S1 and S2).

Associations between COMT genotype and blood pressure measures are also shown in Table 3. There were no associations between COMT genotype and SBP or DBP. In an adjusted model where age was controlled for the results remained unchanged.

**Table 3.** Associations between *COMT* genotype and blood pressure measures (*N* 237**)**

	Β	95 % CI	*P-*value	*β*	95 % CI	*P-*value
Systolic blood pressure						
*G/G* (High activity)	Reference			Reference		
*A/A* (Low activity)	−2⋅930	−7⋅475, 1⋅615	0⋅205	−3⋅321	−7⋅691, 1⋅049	0⋅136
*G/A* (Intermediate activity)	−1⋅823	−6⋅061, 2⋅415	0⋅398	−2⋅240	−6⋅317, 1⋅836	0⋅280
Diastolic blood pressure						
*G/G* (High activity)	Reference			Reference		
*A/A* (Low activity)	−2⋅860	−6⋅196, 0⋅476	0⋅093	−2⋅845	−6⋅191, 0⋅500	0⋅095
*G/A* (Intermediate activity)	−1⋅218	−4⋅329, 1⋅893	0⋅441	−1⋅202	−4⋅323, 1⋅918	0⋅449

CI, confidence interval; *COMT*, catechol-*O*-methyltransferase.

a Crude model: no adjustments were made.

b Adjusted model: adjusted for age.

Subgroup analyses revealed no significant interaction between *COMT* genotype and SBP or DBP measures with age, BMI, adiponectin, leptin, blood pressure category, and current antihypertensive medication use (see Supplementary Tables S3 and S4).

## Discussion

This study explored the potential associations between circulating adiponectin, leptin, and blood pressure measures and additionally examined the potential associations between *COMT* genotype and blood pressure measures in women who are post-menopause. While previous research has shown some evidence of such associations, this study found no association between blood levels of adiponectin and leptin or *COMT* genotype with blood pressure measures in women who were post-menopause.^(9,16,19–22,34)^

Mechanistically, adiponectin plays a role in endothelial function, nitric oxide production and supports the action of anti-inflammatory macrophages which ultimately may affect blood pressure.^(15,16)^ A 5-year, prospective study including both men and women identified an inverse relationship between adiponectin and risk of hypertension; however, exploring these associations in women specifically is important as gender differences, with women usually having higher levels, have been reported.^(13,16–18)^ Additionally, when adiponectin levels have been compared between women who are pre- and post-menopause, it appears menopause may contribute to further increases in circulating adiponectin levels, which may in theory may lead to lower blood pressures and potentially lower risk of hypertension.^(13)^ The findings of this study and other investigations which specifically studied women who are post-menopause have not supported the previously reported inverse relationship found in both men and women however.^(2,16,18–22,43)^ Although not significant, analysis by July *et al.*^(19)^ revealed that women who were post-menopausal (mean age: 60⋅9 ± 9⋅4 years) and hypertensive had lower adiponectin levels compared to women who were normotensive. In the current study, even when data were stratified by blood pressure category, no association was found. Therefore, the results of the current study are consistent with previous research suggesting there is no association with either SBP or DBP values or blood pressure status (normotensive *v.* hypertensive) in this population.^(19–22)^

Leptin is another fat-derived hormone of particular interest in this study. Its primary role in the body is to be a messenger to the brain regarding available energy stores; however, leptin may also impact blood pressure status due to its effect on sympathetic nervous system activation at the renal level.^(14,24–28)^ Many studies have demonstrated a positive correlation between leptin and blood pressure.^(24,29–32)^ This relationship has remained even among non-obese participants suggesting this relationship is independent of weight status.^(29–32)^ Similar to adiponectin, women's leptin levels are generally higher than men's which is also independent of BMI.^(33)^ This gender difference has been associated with a larger proportion of fat mass in women compared to men relative to BMI.^(33)^ Menopause further increases leptin levels, which has been hypothesised to be related to an increase in adipose tissue.^(33)^ Although an association was not found in this study after adjusting for confounding variables, available research suggests there is a relationship between leptin and blood pressure in women who are post-menopause ranging from 50 to 68 years old primarily in relation to SBP.^(20,22,34)^ For example, Olszanecka *et al.*^(22)^ reported a significant positive relationship between leptin and SBP. Furthermore, when analyses were conducted in consideration of blood pressure categorisation by Khokhar *et al.*,^(34)^ leptin was strongly positively correlated with both systolic and diastolic blood pressures in women who were hypertensive and had a normal BMI. A similar pattern was observed for women who were hypertensive and obese.^(34)^ Using a multiple linear regression model, the findings of Khokhar *et al.*^(34)^ also support what is available elsewhere in the literature that a positive relationship exists between leptin and blood pressure, specifically SBP in those who are hypertensive, regardless of weight status. As noted, the associations identified by other researchers were not supported by the findings of this study. We identified a positive association between leptin and SBP in the crude analysis, however, the observed association was no longer statistically significant after adjustment for potential confounders or in stratified analyses.

COMT enzyme activity is dependent upon *COMT* genotype.^(35,36)^ Individuals possessing homozygous variant alleles (*A/A)* and heterozygous variant alleles (*G/A*) show reduced enzymatic activity compared to those with homozygous wild-type alleles (*G/G*) who have higher activity of the COMT enzyme.^(8,9,35,36)^ *COMT* genotype distribution varies based on ethnic backgrounds and the *G/A* variant is found in approximately 40 % of individuals.^(9,38,44,45)^ In individuals of primarily Caucasian descent, the *G/A* variant is found in 40–51 % of the population.^(38,44,45)^ In this study sample, the prevalence of the *G/A* genotype was 46 %.^(38)^ The *A/A* variant is the least common and is found in approximately 20 % of individuals or less.^(9,38,44,45)^ Some studies using primarily Caucasian samples have reported a prevalence of this variant to be up to 21 %, however, the prevalence in this study sample was 31⋅6 %.^(38,44,45)^ As COMT enzyme metabolises specific catecholamines, those with the low activity genotype have a lower capacity for catecholamine metabolism compared to the other genotypes; given the role that catecholamines play in blood pressure regulation a potential for increased blood pressure is plausible in this group.^(7–9,36)^ A significant relationship between *COMTA/A* genotype and higher SBP and DBP was identified in a study completed in Japanese men as compared to those with the *G/A* or *G/G* genotypes.^(9)^ The *A/A* genotype was also associated with a higher risk of hypertension in the study completed by Htun *et al.*^(9)^ This study did not find a significant association between the *COMT* genotype and blood pressure measures; however, this finding may be due to differences in the sample population as participants in the present study were primarily Caucasian. In addition, previous studies have been completed inclusive of both male and female participants.^(8,9)^ To the best of our knowledge, this is the first study exploring the association between *COMT* genotype and blood pressure measures in women who are post-menopause.

There are some limitations to the present study that are worth noting. The first limitation is that the study has a cross-sectional design, therefore, causality cannot be inferred. Secondly, this study is a secondary analysis of a large clinical trial, and the data were not prospectively collected with the aims of the current study in mind. As such the methods used to obtain the data in the MGTT may not have utilised the most ideal methods in consideration of the aims, such as that for blood pressure. Also, as a secondary analysis, this study is limited by the inclusion and exclusion criteria of the parent study. While data were available for many variables of interest within the dataset, we cannot rule out the possibility of residual confounding variables such as energy intake. Additionally, the inclusion and exclusion criteria of the parent study may be a limiting factor for the generalisability of this study as only women who were post-menopause and predominantly White from the Minneapolis-St. Paul metropolitan area were included in the MGTT.^(37,46)^ This study has some strengths including being adequately powered and it utilised a robust statistical approach that accounted for multiple potential confounding variables which have not been done consistently in previous research. As part of the research aim, this study also explored novel variables (adiponectin, leptin, and *COMT* genotype) in a specific population at increased risk of CVD which can also be counted as the strength of the present study.

Overall, this study adds to the available literature exploring the associations between circulating adiponectin and leptin, *COMT* genotype and blood pressure. This study utilised a more robust statistical model than much of the previous research has taken into consideration certain potential confounding variables (BMI, age, WHR, diet components including sodium, caffeine and alcohol consumption and physical activity). The data were also stratified by select participant characteristics to further explore patterns of change within the subgroups. Even with an adequately powered study and additional statistical analyses, this study did not identify significant associations between plasma leptin, plasma adiponectin, *COMT* genotype with blood pressure in women who are post-menopause. Future studies should use a more diverse population to better determine if race or ethnicity has any impact on the associations explored. In addition, this study only looked at baseline data from the MGTT which reflected one point in time, as with some existing research; future studies could utilise a prospective design to further explore the potential associations over time.

## Supporting information

Green et al. supplementary material Green et al. supplementary material

## Supplementary material

The supplementary material for this article can be found at https://doi.org/10.1017/jns.2023.75.
