# Supplementary material for: Exploration of plasma adiponectin, leptin, and COMT genotype on blood pressure among women who are post-menopause
Source: J Nutr Sci. 2023 Sep 19;12:e100. doi: 10.1017/jns.2023.75 (PMC10523287; doi:10.1017/jns.2023.75)
Supplement: Green et al. supplementary material [file S2048679023000757sup001.docx]

| **Table S1. Associations between circulating concentrations of adiponectin and leptin and systolic blood pressure by age, BMI, *COMT* genotype, blood pressure category and current antihypertensive medication use (N=237)** | | | | | | | | | |
| --- | --- | --- | --- | --- | --- | --- | --- | --- | --- |
|  | **Adiponectin** | | |  | | **Leptin** | | | |
|  | **β** | **95% CI** | ***P_int_*** | |  | | **β** | **95% CI** | ***P_int_*** |
| **Age (y)^1^** |  |  | 0.581 | |  | |  |  | 0.780 |
| 50 – 59 | Reference |  |  | |  | | Reference |  |  |
| 60 – 70 | 4.931 | -0.247, 10.110 |  | |  | | 5.328 | -0.920, 11.576 |  |
|  |  |  |  | |  | |  |  |  |
| **Body mass index (kg/m^2^)^2^** |  |  | 0.308 | |  | |  |  | 0.800 |
| Normal (18.5 to 24.9) | Reference |  |  | |  | | Reference |  |  |
| Overweight (25.0 to 29.9) | 6.239 | 1.197, 11.281 |  | |  | | 5.889 | 0.835, 10.942 |  |
| Obese (≥30) | 10.302 | 4.664, 15.939 |  | |  | | 8.847 | 2.747, 14.947 |  |
|  |  |  |  | |  | |  |  |  |
| ***COMT* genotype^3^** |  |  | 0.458 | |  | |  |  | 0.499 |
| *G/G* (High activity) | Reference |  |  | |  | | Reference |  |  |
| *A/A* (Low activity) | -3.222 | -7.507, 1.063 |  | |  | | -3.244 | -7.517, 1.028 |  |
| *G/A* (Intermediate activity) | -1.643 | -5.675, 2.389 |  | |  | | -1.721 | -5.724, 2.282 |  |
|  |  |  |  | |  | |  |  |  |
| **Blood pressure category^3,4^** |  |  | 0.890 | |  | |  |  | 0.621 |
| Normotensive | Reference |  |  | |  | | Reference |  |  |
| Elevated blood pressure | 12.772 | 10.102, 15.442 |  | |  | | 12.799 | 10.127, 15.470 |  |
| Hypertensive | 23.129 | 20.892, 25.366 |  | |  | | 23.104 | 20.865, 25.343 |  |
|  |  |  |  | |  | |  |  |  |
| **Current antihypertensive medication use^3^** |  |  | 0.502 | |  | |  |  | 0.311 |
| No | Reference |  |  | |  | | Reference |  |  |
| Yes | 9.612 | 4.817, 14.406 |  | |  | | 13.724 | 7.250, 20.197 |  |
| Abbreviations: BMI; body mass index; CI, confidence interval; *COMT*, catechol-O-methyltransferase; DBP, diastolic blood pressure; SBP, systolic blood pressure. | | | | | | | | | |
| ^1^ Model adjusted for BMI and physical activity. ^2^ Model adjusted for age and physical activity. ^3^ Model adjusted for age, BMI and physical activity.  ^4^ Normotensive is defined as SBP<120 mmHg and DBP<80 mmHg; elevated blood pressure as SBP=120-129 mmHg and DBP<80 mmHg; and hypertensive as SBP≥130 mmHg or DBP≥80 mmHg. | | | | | | | | | |

| **Table S2. Associations between circulating concentrations of adiponectin and leptin and diastolic blood pressure measures by age, BMI, *COMT* genotype, blood pressure category and current antihypertensive medication use (N=237)** | | | | | | | |
| --- | --- | --- | --- | --- | --- | --- | --- |
|  | **Adiponectin** | | |  | **Leptin** | | |
|  | **β** | **95% CI** | ***P_int_*** |  | **β** | **95% CI** | ***P_int_*** |
| **Age (y)^1^** |  |  | 0.163 |  |  |  | 0.132 |
| 50 – 59 | Reference |  |  |  | Reference |  |  |
| 60 – 70 | -3.217 | -7.145, 0.711 |  |  | -4.144 | -8.884, 0.596 |  |
|  |  |  |  |  |  |  |  |
| **Body mass index (kg/m^2^)^2^** |  |  | 0.375 |  |  |  | 0.200 |
| Normal (18.5 to 24.9) | Reference |  |  |  | Reference |  |  |
| Overweight (25.0 to 29.9) | 3.238 | -0.656, 7.132 |  |  | 3.166 | -0.752, 7.083 |  |
| Obese (≥30) | 6.167 | 1.813, 10.521 |  |  | 6.012 | 1.283, 10.741 |  |
|  |  |  |  |  |  |  |  |
| ***COMT* genotype^3^** |  |  | 0.818 |  |  |  | 0.926 |
| *G/G* (High activity) | Reference |  |  |  | Reference |  |  |
| *A/A* (Low activity) | -2.771 | -6.068, 0.525 |  |  | -2.815 | -6.110, 0.480 |  |
| *G/A* (Intermediate activity) | -0.682 | -3.783, 2.420 |  |  | -0.775 | -3.862, 2.312 |  |
|  |  |  |  |  |  |  |  |
| **Blood pressure category^3,4^** |  |  | 0.999 |  |  |  | 0.557 |
| Normotensive | Reference |  |  |  | Reference |  |  |
| Elevated blood pressure | 4.747 | 2.238, 7.257 |  |  | 4.695 | 2.186, 7.203 |  |
| Hypertensive | 14.792 | 12.689, 16.895 |  |  | 14.850 | 12.747, 16.952 |  |
|  |  |  |  |  |  |  |  |
| **Current antihypertensive medication use^3^** |  |  | 0.730 |  |  |  | 0.758 |
| No | Reference |  |  |  | Reference |  |  |
| Yes | 6.194 | 2.547, 9.841 |  |  | 7.325 | 2.384, 12.265 |  |
| Abbreviations: BMI; body mass index; *COMT*, catechol-O-methyltransferase; CI, confidence interval; DBP, diastolic blood pressure; SBP, systolic blood pressure. | | | | | | | |
| ^1^ Model adjusted for BMI and physical activity. ^2^ Model adjusted for age and physical activity. ^3^ Model adjusted for age, BMI and physical activity.  ^4^ Normotensive is defined as SBP<120 mmHg and DBP<80 mmHg; elevated blood pressure as SBP=120-129 mmHg and DBP<80 mmHg; and hypertensive as SBP≥130 mmHg or DBP≥80 mmHg. | | | | | | | |

| **Table S3. Associations between *COMT* genotype and systolic blood pressure by age, BMI, circulating concentrations of plasma adiponectin and leptin, blood pressure category and current antihypertensive medication use (N=237)** | | | | | | | | | |
| --- | --- | --- | --- | --- | --- | --- | --- | --- | --- |
|  | ***G/G* (High activity)** | |  | ***A/A* (Low activity)** | |  | ***G/A* (Intermediate activity)** | | ***P_int_*** |
|  | **β** | **95% CI** |  | **β** | **95% CI** |  | **β** | **95% CI** |  |
| **Age (y)** |  |  |  |  |  |  |  |  | 0.573 |
| 50 – 59 | Reference |  |  | -2.003 | -8.993, 4.987 |  | -2.631 | -9.232, 3.971 |  |
| 60 – 70 | Reference |  |  | -4.479 | -10.095, 1.137 |  | -2.066 | -7.239, 3.106 |  |
|  |  |  |  |  |  |  |  |  |  |
| **Body mass index (kg/m^2^)^1^** |  |  |  |  |  |  |  |  | 0.978 |
| Normal (18.5 to 24.9) | Reference |  |  | 4.258 | -8.748, 17.265 |  | 2.213 | -10.299, 14.725 |  |
| Overweight (25.0 to 29.9) | Reference |  |  | -2.469 | -8.211, 3.274 |  | -1.838 | -7.168, 3.492 |  |
| Obese (≥30) | Reference |  |  | -7.382 | -15.064, 0.300 |  | -4.460 | -11.749, 2.830 |  |
|  |  |  |  |  |  |  |  |  |  |
| **Adiponectin (µg/mL)^1^** |  |  |  |  |  |  |  |  | 0.072 |
| 1.87 - 6.29 | Reference |  |  | -4.347 | -10.308, 1.614 |  | -6.104 | -11.568, -0.640 |  |
| 6.30 - 70.48 | Reference |  |  | -1.060 | -7.540, 5.421 |  | 2.688 | -3.445, 8.821 |  |
|  |  |  |  |  |  |  |  |  |  |
| **Leptin (µg/L)^1^** |  |  |  |  |  |  |  |  | 0.691 |
| 5.15 - 31.29 | Reference |  |  | -3.985 | -10.389, 2.419 |  | -4.196 | -10.323, 1.931 |  |
| 31.30 - 161.51 | Reference |  |  | -2.539 | -8.630, 3.551 |  | -0.390 | -5.928, 5.148 |  |
|  |  |  |  |  |  |  |  |  |  |
| **Blood Pressure Category^1,2^** |  |  |  |  |  |  |  |  | 0.886 |
| Normotensive | Reference |  |  | -0.077 | -4.050, 3.897 |  | 2.140 | -1.621, 5.901 |  |
| Elevated Blood Pressure | Reference |  |  | -0.442 | -2.667, 1.783 |  | 0.393 | -1.506, 2.292 |  |
| Hypertensive | Reference |  |  | -3.467 | -8.115, 1.181 |  | -2.968 | -7.346, 1.410 |  |
|  |  |  |  |  |  |  |  |  |  |
| **Current Antihypertensive Medication Use^1^** |  |  |  |  |  |  |  |  | 0.618 |
| No | Reference |  |  | -3.604 | -8.481, 1.272 |  | -2.305 | -6.875, 2.264 |  |
| Yes | Reference |  |  | -2.105 | -9.634, 5.424 |  | -3.993 | -10.919, 2.932 |  |
| Abbreviations: BMI, body mass index; CI, confidence intervals; *COMT*, catechol-O-methyltransferase; DBP, diastolic blood pressure; SBP, systolic blood pressure. | | | | | | | | | |
| ^1^ Model adjusted for age.; ^2^ Normotensive is defined as SBP<120 mmHg and DBP<80 mmHg; elevated blood pressure as SBP=120-129 mmHg and DBP<80 mmHg; and hypertensive as SBP≥130 mmHg or DBP≥80 mmHg. | | | | | | | | | |

| **Table S4. Associations between *COMT* genotype and diastolic blood pressure by age, BMI, circulating concentrations of plasma adiponectin and leptin, blood pressure category and current antihypertensive medication use^1^ (N=237)** | | | | | | | | | | |
| --- | --- | --- | --- | --- | --- | --- | --- | --- | --- | --- |
|  | ***G/G* (High activity)** |  |  | ***A/A (Low activity)*** | |  | ***G/A (Intermediate activity)*** | |  | ***P_int_*** |
|  | **β** | **95% CI** |  | **β** | **95% CI** |  | **β** | **95% CI** |  |  |
| **Age (y)** |  |  |  |  |  |  |  |  |  | 0.671 |
| 50 – 59 | Reference |  |  | -1.237 | -6.258, 3.783 |  | 0.111 | -4.630, 4.852 |  |  |
| 60 – 70 | Reference |  |  | -4.619 | -9.174, -0.064 |  | -2.336 | -6.532, 1.859 |  |  |
|  |  |  |  |  |  |  |  |  |  |  |
| **Body mass index (kg/m^2^)^2^** |  |  |  |  |  |  |  |  |  | 0.679 |
| Normal (18.5 to 24.9) | Reference |  |  | -1.495 | -12.086, 9.096 |  | -0.278 | -10.466, 9.910 |  |  |
| Overweight (25.0 to 29.9) | Reference |  |  | -3.210 | -7.712, 1.291 |  | -1.825 | -6.003, 2.353 |  |  |
| Obese (≥30) | Reference |  |  | -2.154 | -7.925, 3.617 |  | 0.161 | -5.314, 5.637 |  |  |
|  |  |  |  |  |  |  |  |  |  |  |
| **Adiponectin (µg/mL)^2^** |  |  |  |  |  |  |  |  |  | 0.052 |
| 1.87 - 6.29 | Reference |  |  | -4.104 | -8.743, 0.536 |  | -4.387 | -8.640, -0.135 |  |  |
| 6.30 - 70.48 | Reference |  |  | -1.149 | -6.028, 3.731 |  | 2.418 | -2.200, 7.036 |  |  |
|  |  |  |  |  |  |  |  |  |  |  |
| **Leptin (µg/L)^2^** |  |  |  |  |  |  |  |  |  | 0.625 |
| 5.15 - 31.29 | Reference |  |  | -4.084 | -8.747, 0.579 |  | -2.559 | -7.020, 1.902 |  |  |
| 31.30 - 161.51 | Reference |  |  | -1.470 | -6.385, 3.444 |  | 0.193 | -4.276, 4.661 |  |  |
|  |  |  |  |  |  |  |  |  |  |  |
| **Blood pressure category^2,3^** |  |  |  |  |  |  |  |  |  | 0.514 |
| Normotensive | Reference |  |  | -2.964 | -6.897, 0.969 |  | -0.078 | -3.801, 3.644 |  |  |
| Elevated blood pressure | Reference |  |  | -1.546 | -6.447, 3.354 |  | -0.365 | -4.548, 3.817 |  |  |
| Hypertensive | Reference |  |  | -2.100 | -5.999, 1.799 |  | -0.584 | -4.256, 3.089 |  |  |
|  |  |  |  |  |  |  |  |  |  |  |
| **Current antihypertensive medication use^2^** |  |  |  |  |  |  |  |  |  | 0.659 |
| No | Reference |  |  | -3.722 | -7.229, -0.215 |  | -1.417 | -4.703, 1.868 |  |  |
| Yes | Reference |  |  | -0.016 | -6.877, 6.844 |  | -1.913 | -8.223, 4.398 |  |  |
| Abbreviations: BMI, body mass index; CI, confidence intervals; COMT, catechol-O-methyltransferase; DBP, diastolic blood pressure; SBP, systolic blood pressure. | | | | | | | | | | |
| ^1^ *G/G (High activity)COMT* genotype was used as reference.  ^2^ Model adjusted for age.  ^3^ Normotensive is defined as SBP<120 mmHg and DBP<80 mmHg; elevated blood pressure as SBP=120-129 mmHg and DBP<80 mmHg; and hypertensive as SBP≥130 mmHg or DBP≥80 mmHg. | | | | | | | | | | |
